# Single-Stage Medial Meniscus Ramp Repair, Lateral Meniscus Root Repair, Revision Anterior Cruciate Ligament Reconstruction and Lateral Extra-Articular Tenodesis

**DOI:** 10.1016/j.eats.2025.103578

**Published:** 2025-05-02

**Authors:** Samundeeswari Saseendar, Salim AL. Habsi, Ying Ren Mok, Yee Han Dave Lee

**Affiliations:** Division of Sports, Shoulder and Elbow Surgery, Department of Orthopedic Surgery, National University Hospital, Singapore

## Abstract

Revision anterior cruciate ligament reconstruction (ACLR) is being performed increasingly as a result of the growing number of primary ACLR failures. The combined lesions of medial meniscus ramp lesions and lateral meniscus posterior root tears are being observed among many patients who undergo revision ACLR. This combination of meniscus injuries is referred to as "the new terrible triad." Various studies suggest that failing to address these meniscus injuries during primary ACLR can increase the risk of anterior cruciate ligament graft failure. In this technique report, we present our systematic approach to manage medial meniscus ramp lesions and lateral meniscus posterior root tears in revision ACLR.

The rate of revision anterior cruciate ligament reconstruction (ACLR) ranges from 4% to 25%.^1^^,^^2^ Graft failures are caused by various factors, with the most common being persistent knee instability after ACLR.^2^ Magosch et al.^3^ reported that medial meniscus ramp lesions (MMRLs) or lateral meniscus posterior root tears (LMPRTs) are present in more than one-third of primary and revision ACLR. The presence of both MMRL and LMPRT injuries are seen in up to 8% of primary ACLR.^4^^,^^5^ This combination of injuries has been termed as the “new terrible triad” of the knee.^6^ These combined injuries impact knee biomechanics by increasing anterior tibial translation and external rotation.^4^^,^^7^ If left untreated, these injuries can significantly increase the risk of anterior cruciate ligament (ACL) graft failure by up to 4.9 times.^2^^,^^4^^,^^7^

The complexity of revision ACLR increases with these combined meniscus injuries, makes surgical planning in the treatment of these injuries critical. We outline our systematic management approach to address all these injuries in revision ACLR.

### Assessment

The preoperative assessment of all patients scheduled to undergo revision ACL should include a thorough physical examination to evaluate for high-grade knee laxity. This is complimented with an examination under anesthesia to confirm the presence of a high-grade Lachman test and an explosive pivot shift tests. These indicate the possibility of significant meniscus injuries such as MMRL and LMPRT.^8^

The radiographs of the knee (Fig 1) and computed tomography scan are used to assess the tunnel position and diameter (Fig 2). In this case, the previous femur and tibial tunnels were deemed to be acceptable with some tunnel widening (<1.1 cm in size). Hence, a single-stage revision ACLR using the previous femur and tibial tunnels was planned. A careful study of the magnetic resonance imaging should be performed to examine the lateral meniscus posterior root (LMPR) and medial meniscocapsular junction (Fig 3).

**Fig 1:**
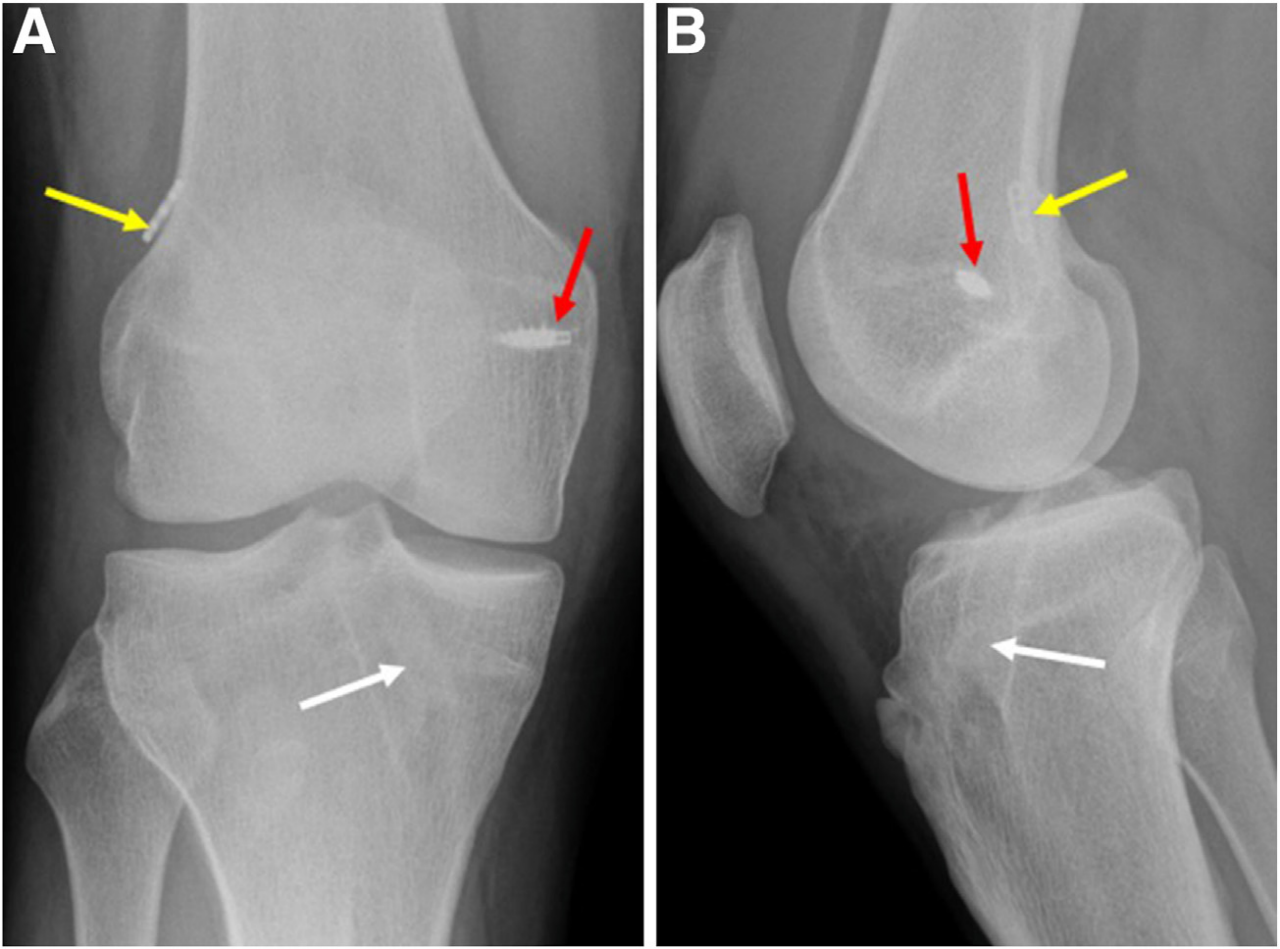
(A and B) Anteroposterior and lateral right knee preoperative radiographs showing previous ACL tunnel position in the tibia (white arrow), femur button position (yellow arrow), and previous MCL repair screw position (red arrow). (ACL, anterior cruciate ligament; MCL, medial collateral ligament)

**Fig 2:**
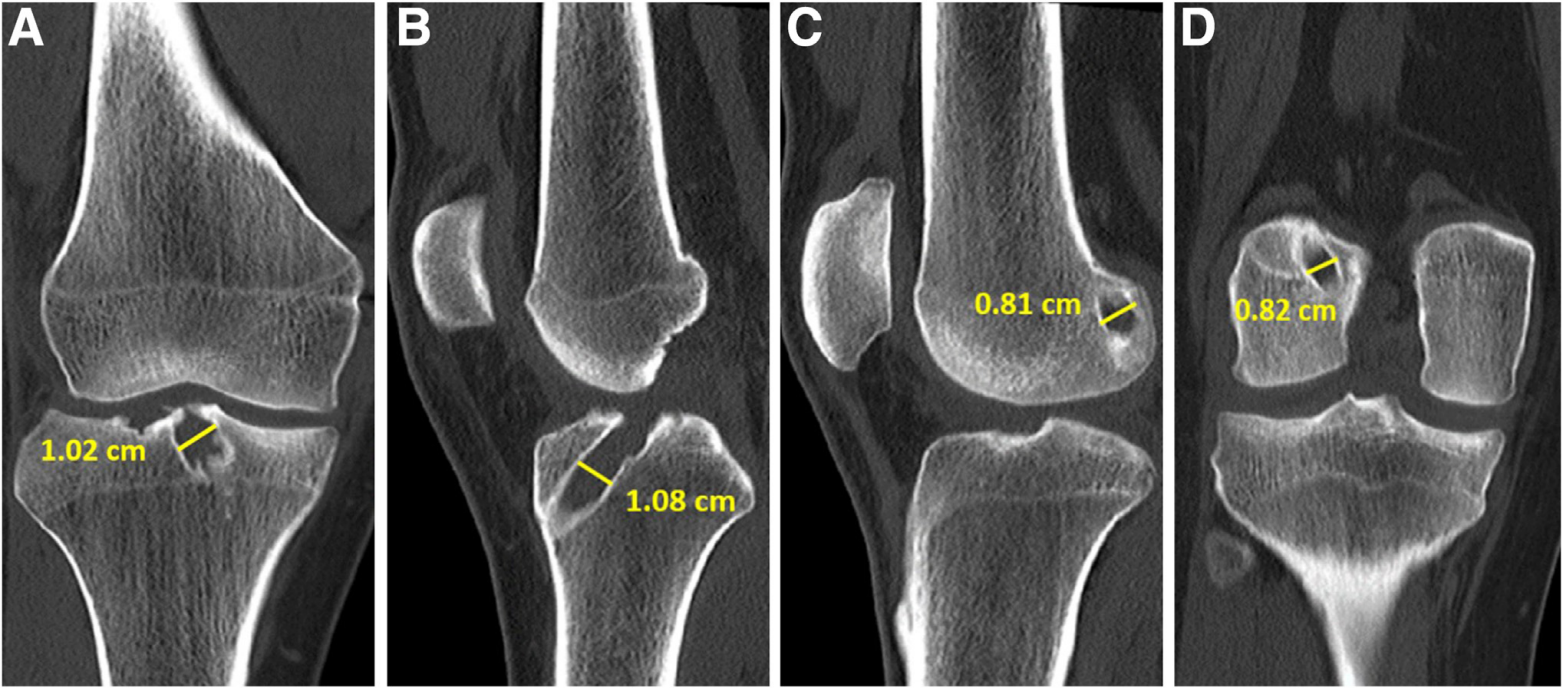
Computed tomography scans of the right knee in a patient with new terrible triad and ACL graft rupture. The scans show the measurement of the dimensions and position of the previous femur and tibia tunnels. (A) Coronal cut of tibia tunnel. (B) Sagittal cut of tibia tunnel. (C) Sagittal cut of femur tunnel. (D) Coronal cut of femur tunnel. (ACL, anterior cruciate ligament)

**Fig 3:**
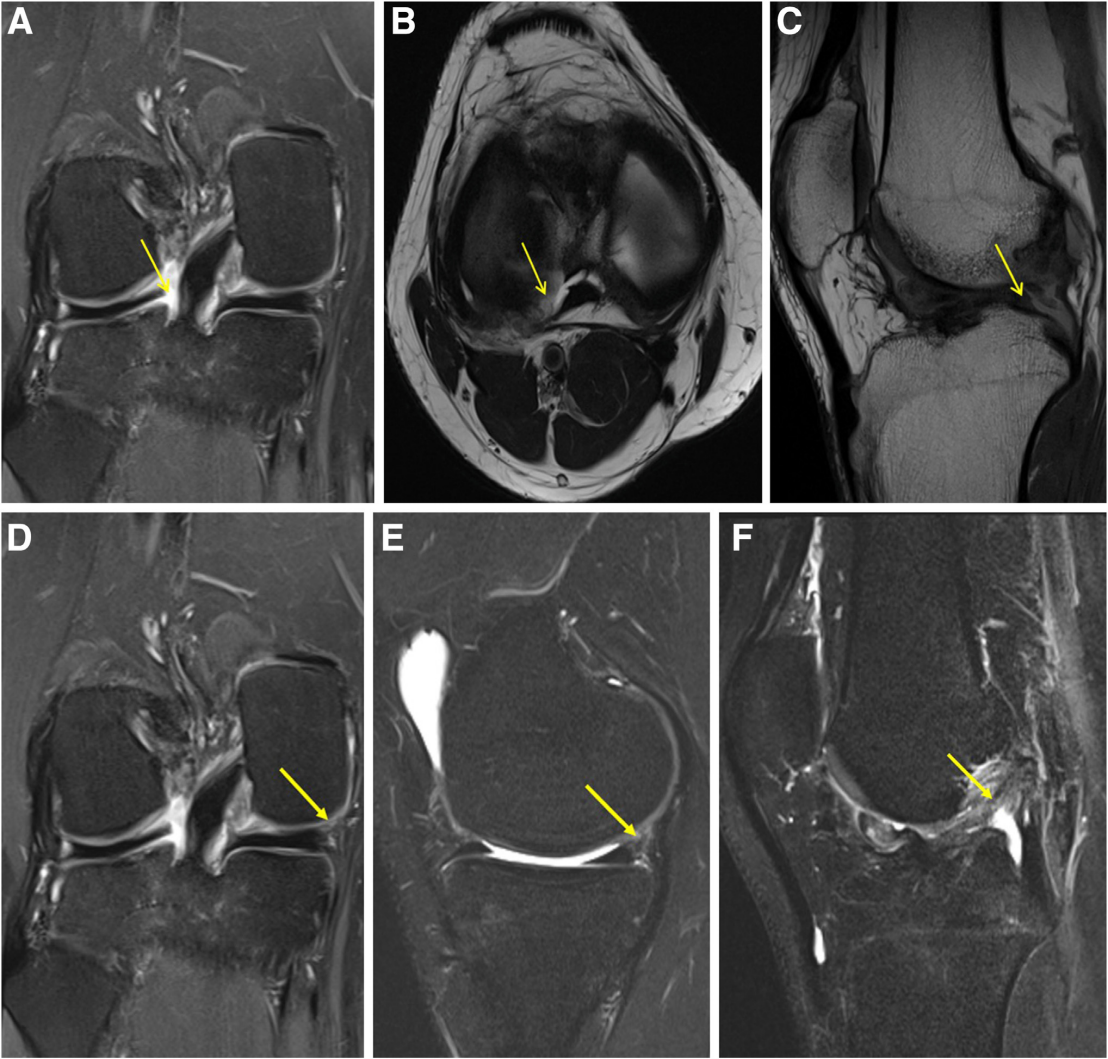
MRI of right knee in a patient with new terrible triad and ACL graft rupture. (A) Coronal T2 PD FS shows increased hyperintensity signals (yellow arrow) suggestive of lateral meniscus posterior root tear. (B) Axial T1 PD FS shows increased hyperintensity signals (yellow arrow) suggestive of lateral meniscus posterior root tear. (C) Sagittal T1 PD FS shows increased hyperintense signal (yellow arrow) suggestive of lateral meniscus root tear, also known as “ghost sign.” (D) Coronal T2 PD FS shows increased hyperintense signals in the posterior horn of medial meniscus suggestive of medial meniscus ramp lesion (yellow arrow). (E) Sagittal T2 PD FS shows increased hyperintense signals in the posterior horn of the medial meniscus suggestive of medial meniscus ramp lesion (yellow arrow). (F) T1 PD FS shows a complete tear of the ACL graft (yellow arrow). (ACL, anterior cruciate ligament; FS, fat saturation sequence; MRI, magnetic resonance imaging; PD, proton density.)

## Surgical Technique

**Fig 4:**
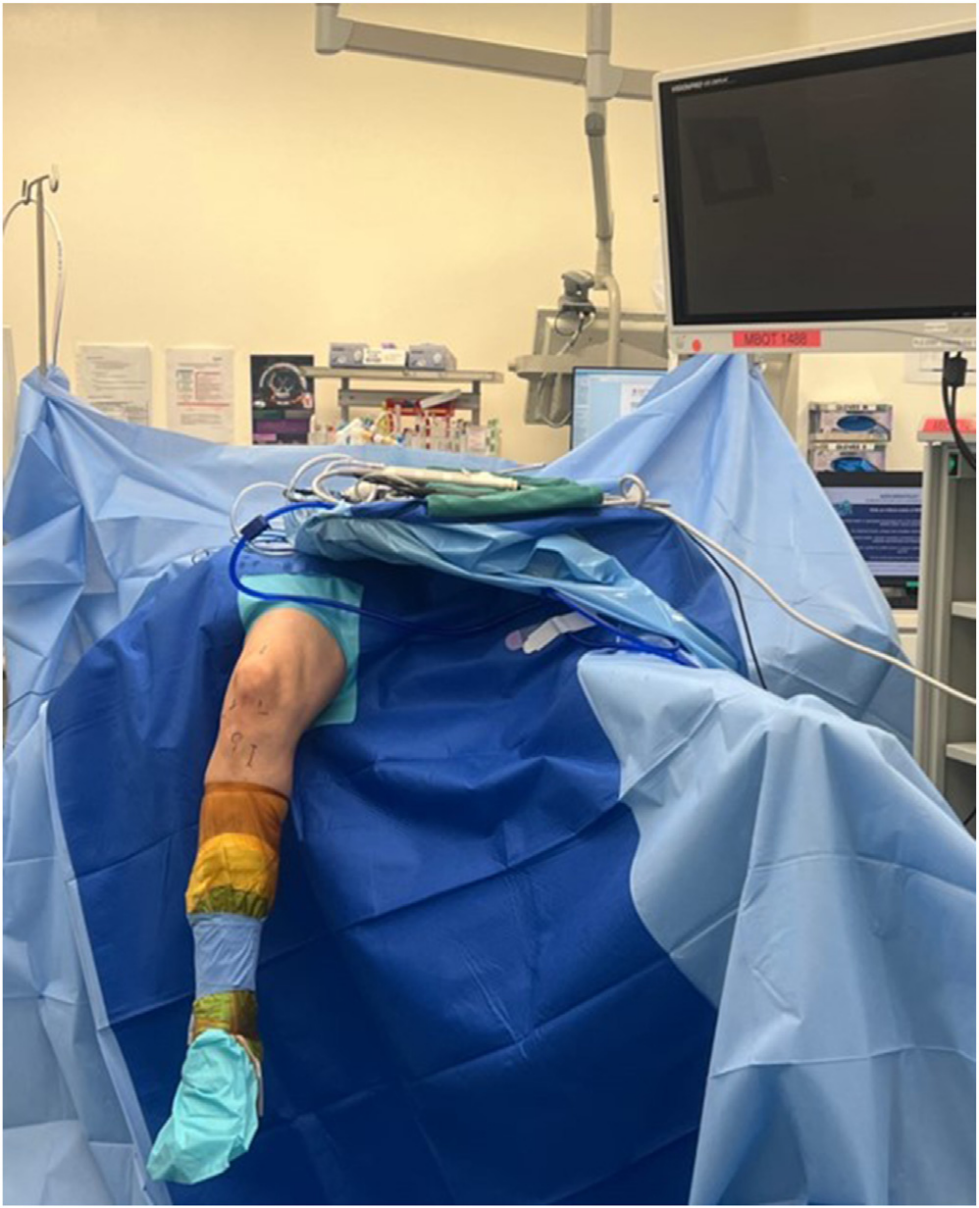
Patient positioning during right knee arthroscopic revision ACLR combined with ramp tear repair and LMPRT repair. The patient is positioned supine on the operating table under general anesthesia with the leg suspended. The nonoperated left lower limb is placed in a stirrup leg holder. A standard waterproof arthroscopy drape is used. (ACLR, anterior cruciate ligament reconstruction; LMPRT, lateral meniscus posterior root tear.)

The patient is positioned supine on the operating table under general anesthesia with the leg suspended. A well-padded tourniquet is applied proximally on the thigh. The opposite leg is placed in a stirrup leg holder (Fig 4). A near full-thickness size 11-mm width quadriceps tendon graft was harvested and prepared with cortical fixation on both the femur and tibia.

### Arthroscopic Evaluation

Anterolateral and anteromedial knee portals are established for arthroscopic evaluation using a 30° knee arthroscope. In patients who undergo revision ACL, careful examination of the medial meniscus and lateral meniscus needs to be performed. The systematic approach of our surgical technique is described in (Video 1).

### Medial Meniscus Ramp Repair

**Fig 5:**
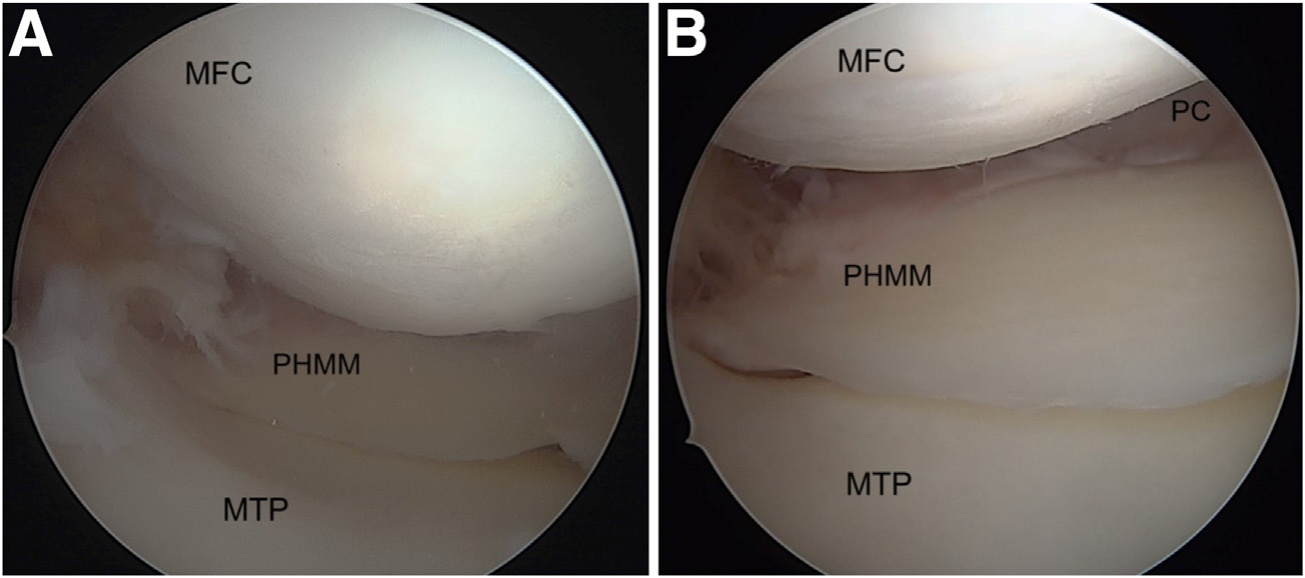
Arthroscopic pictures of right knee in complete extension with valgus stress given by the assistant (patient in supine position, view from anterolateral portal). (A) Medial meniscus posterior horn before pie-crusting. (B) Medial meniscus posterior horn after pie-crusting. The medial compartment space has increased allowing better visualization of the PHMM. (MFC, medial femoral condyle; MTP, medial tibial plateau; PC, posterior capsule; PHMM, posterior horn of medial meniscus.)

**Fig 6:**
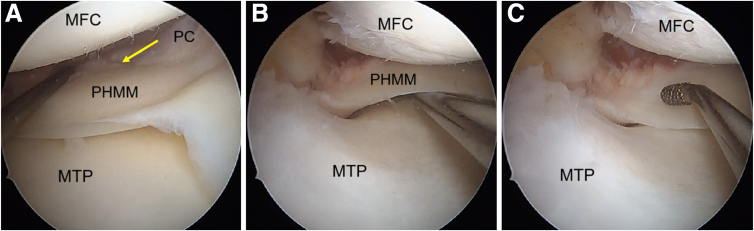
Arthroscopic pictures of right knee medial compartment, (patient in supine position, view from anterolateral portal). (A) Probing the upper surface to look for the meniscocapsular separation, reveals the presence of chronic ramp lesion (yellow arrow). (B) Probing the undersurface to look for the meniscocapsular separation, indicates a tear of the meniscotibial ligament. (C) Meniscus diamond rasp is used to clean and freshen the torn ends of the meniscus. (MFC, medial femoral condyle; MTP, medial tibial plateau; PC, posterior capsule; PHMM, posterior horn of medial meniscus.)

Needle pie-crusting of the medial collateral ligament (MCL) is performed with the knee in near full extension with a valgus force applied. The needle is inserted just distal to the femoral insertion of the MCL. An adequate release allows good visualisation of the posterior horn of the medial meniscus and the posterior capsule (Fig 5). A probe is used to identify the meniscocapsular separation and the increased anterior translation of the posterior horn of medial meniscus (Fig 6 A and B). Viewing the meniscocapsular junction through the intercondylar notch can help identify the meniscocapsular separation, which can further be accentuated by bringing the knee into flexion and rotating the tibia.

**Fig 7:**
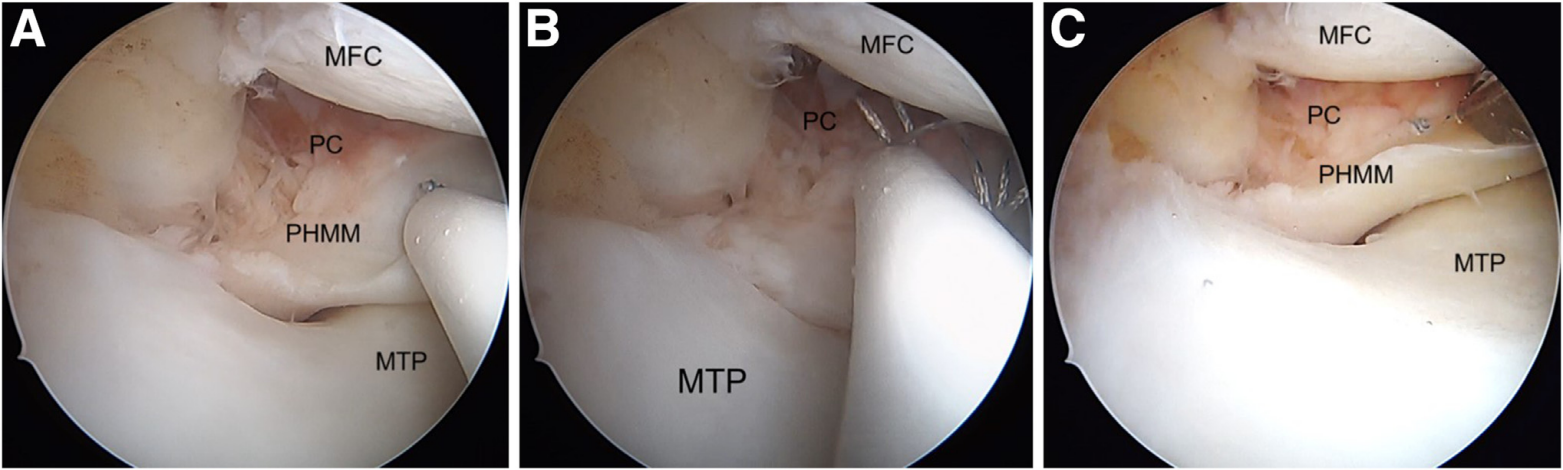
Arthroscopic pictures of right knee medial compartment, (patient in supine position, view from anterolateral portal). The ramp lesion is sutured using all-inside devices. (A) The passage of an all-inside device taking the first bite at the body of the PHMM to deploy the first implant. (B) The second all-inside device implant deployed into the capsule. (C) Tightening of the vertical suture configuration is done using the meniscus suture cutter. (MFC, medial femoral condyle; MTP, medial tibial plateau; PHMM, posterior horn of medial meniscus; PC, posterior capsule.)

**Fig 8:**
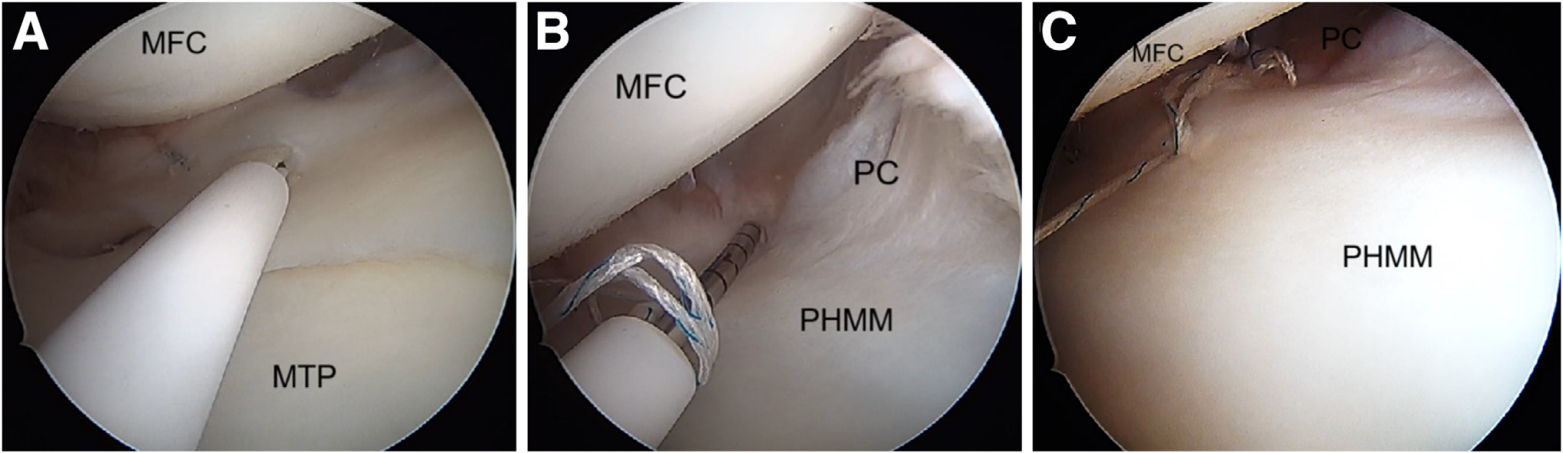
Arthroscopic pictures of right knee medial compartment (patient in supine position, view from anterolateral portal). The steps are repeated in the same fashion until we close the meniscocapsular separation. (A) The passage of an all-inside device taking the first bite at the body of the PHMM to deploy the first implant. (B) The second all-inside device implant deployed into the capsule. (C) Tightening of the vertical suture configuration is done using the meniscus suture cutter. (MFC, medial femoral condyle; MTP, medial tibial plateau; PC, posterior capsule; PHMM, posterior horn of medial meniscus.).

The edges of the lesion are refreshed using an arthroscopic rasp (Fig 6C). The meniscus repair is achieved by placing meniscus sutures at the superior and inferior portion of the medial meniscus and adjacent posterior capsule. The first stitch goes through the superior surface of medial meniscus (Fig 7A), and the second stitch is passed through the capsule (Fig 7B) using a curved FAST-FIX 360 (Smith & Nephew London, England) to achieve a vertical suture configuration (Fig 7C). The steps are repeated to place another set of vertical stitches (Fig 8), until the closure of meniscocapsular separation is observed.

**Fig 9:**
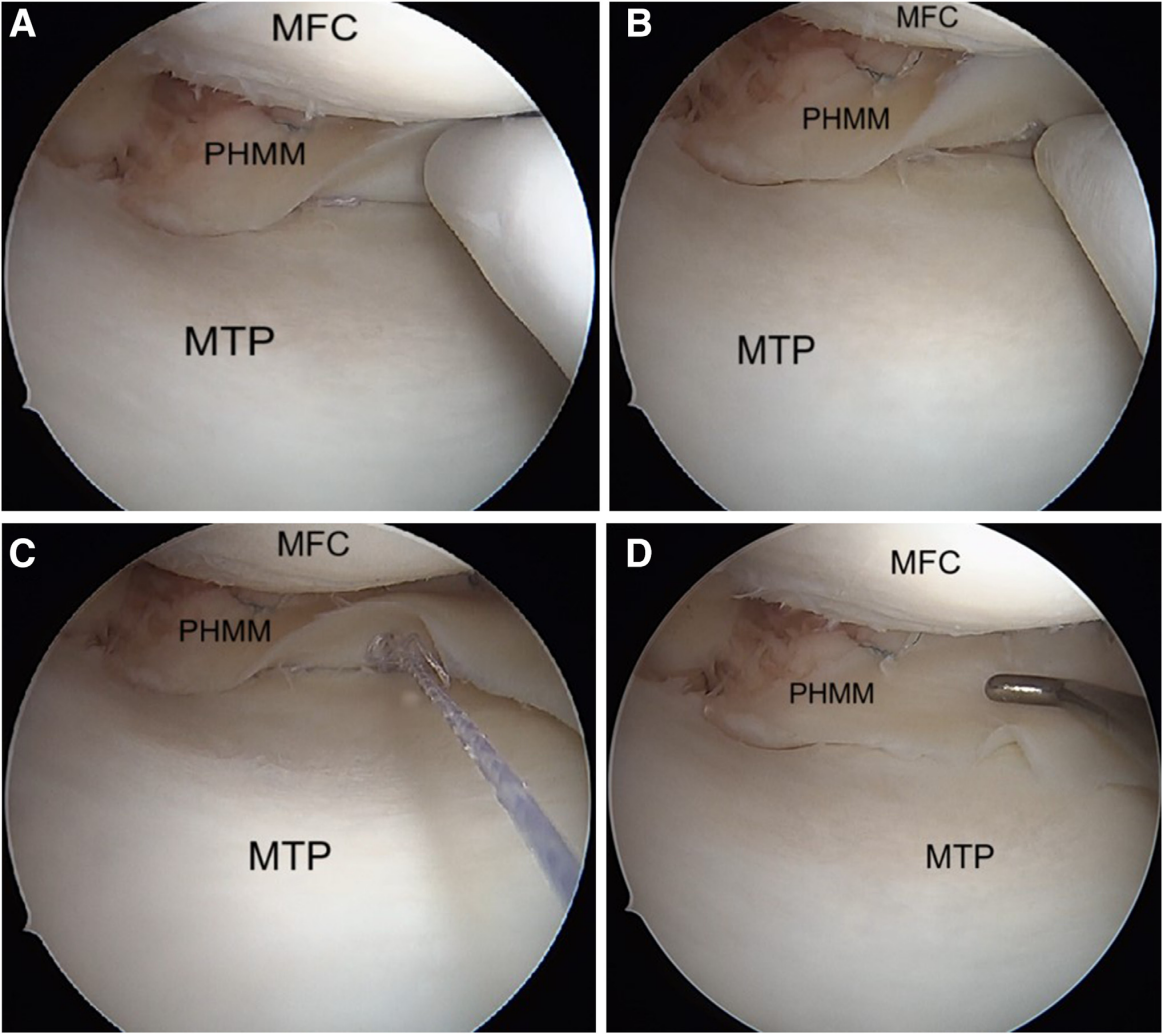
Arthroscopic pictures of right knee medial compartment, (patient in supine position, view from anterolateral portal). The images show the repair of the undersurface tear of the ramp lesion in a vertical suture configuration. (A) Using an all-inside device, we take the first stitch at the inferior surface of the PHMM. (B) The second stitch is passed through the posterior capsule. (C) The final tightening restores the normal anatomy of the PHMM. (D) After final repair of ramp lesion. (MFC, medial femoral condyle; MTP, medial tibial plateau; PHMM, posterior horn of medial meniscus.)

This is repeated on the inferior surface to place a vertical stitch on the undersurface of the meniscus; again, with first stitch through the inferior surface of medial meniscus (Fig 9A), and the second stitch is passed through the capsule (Fig 9B) using a reverse curved FAST-FIX 360. This repairs the meniscotibial ligament (Fig 9C). The sutures are placed sequentially on the superior and inferior aspects of the meniscus until stable and balanced repair of the ramp lesion is achieved (Fig 9D).

### Transtibial Pull-Out Repair of LMPRT

**Fig 10:**
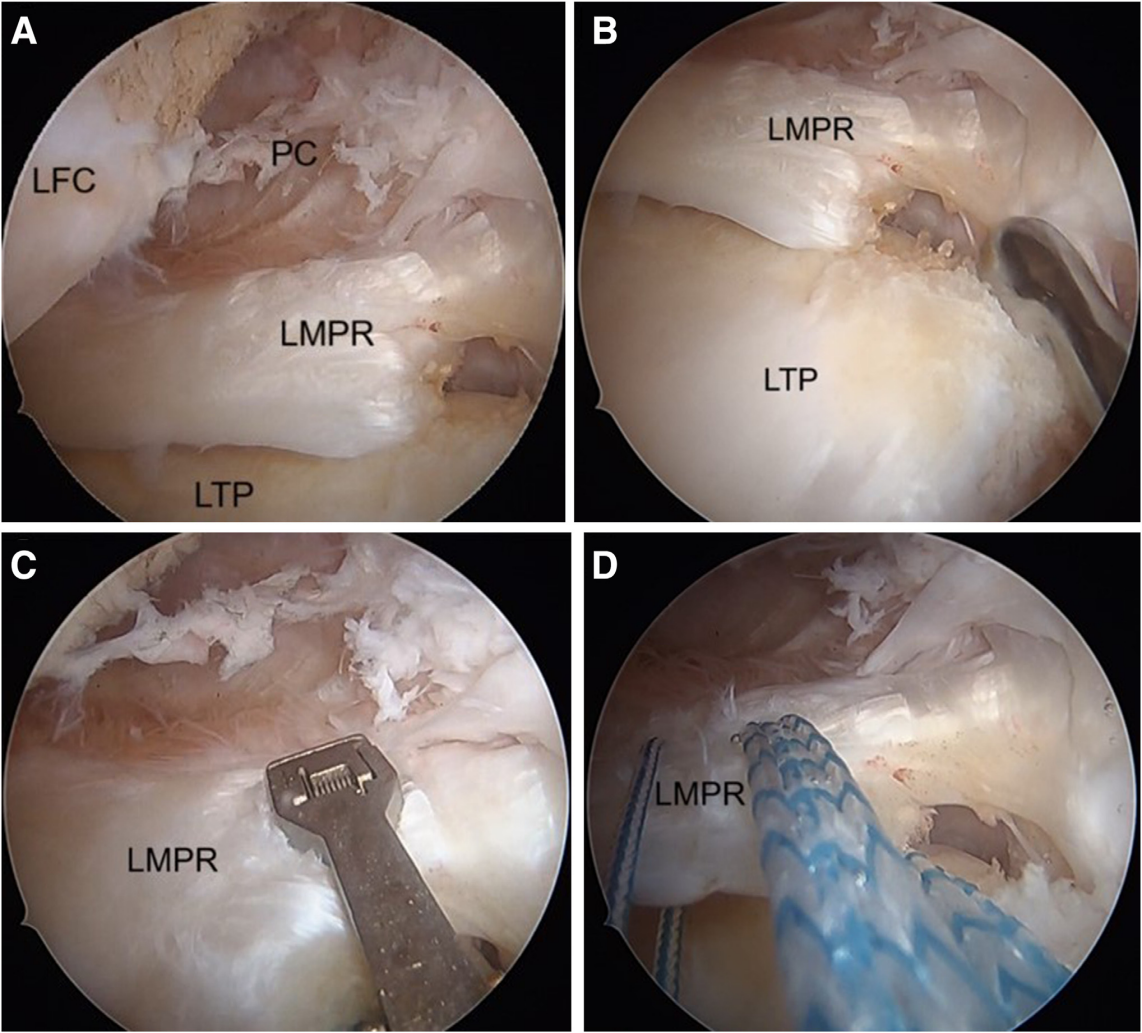
Arthroscopic pictures of right knee showing LMPR repair done in figure-of-four position (visualizing from the anterolateral portal and working from the anteromedial portal). (A) The LMPR tear seen during diagnostic arthroscopy. (B) A closed curette is used to prepare the bed of the root attachment. (C) Placing the antegrade suture passer to pass a suture tape and FiberWire through the LMPR. (D) The tape and FiberWire are passed through the substance of the LMPR, the FiberWire 5 mm lateral to the suture tape in the LMPR. (LFC, lateral femoral condyle; LMPR, lateral meniscus posterior root; LTP, lateral tibial plateau; PC, posterior capsule.)

The lateral compartment is carefully evaluated, with the LMPRT and its footprint is identified (Fig 10A). The scarring between the posterior horn and the capsule is released and the footprint is prepared using a curette (Fig 10B). Suture tape (UltraTape #2; Smith & Nephew) is passed through the lateral meniscus root at approximately 3 mm lateral to the root using an antegrade suture-passing device (Knee Scorpion; Arthrex, Naples, FL) (Fig 10C). A high-strength suture (ULTRABRAID #2; Smith & Nephew) is passed 5 mm lateral to the suture tape (Fig 10D). The tape and suture passage is performed in robust meniscus tissue, close to the lateral meniscus capsule junction.

**Fig 11:**
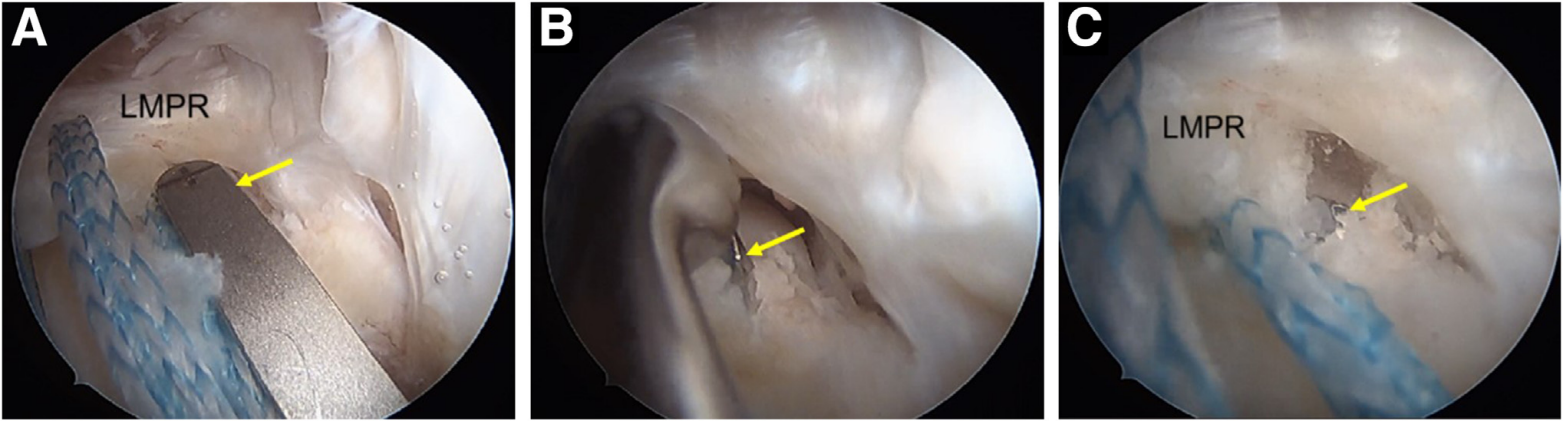
Arthroscopic pictures of right knee showing LMPR repair (patient in supine position, right limb in figure-of-four position, view from anterolateral portal). (A) The aiming guide (yellow arrow) of the lateral meniscus root is placed anatomically. (B) A closed curette is used to protect the drill guide (yellow arrow) from overpenetrating. (C) Tibial tunnel drilled at the footprint of the LMPR with a 4.5-mm reamer (yellow arrow). (LMPR, lateral meniscus posterior root.)

**Fig 12:**
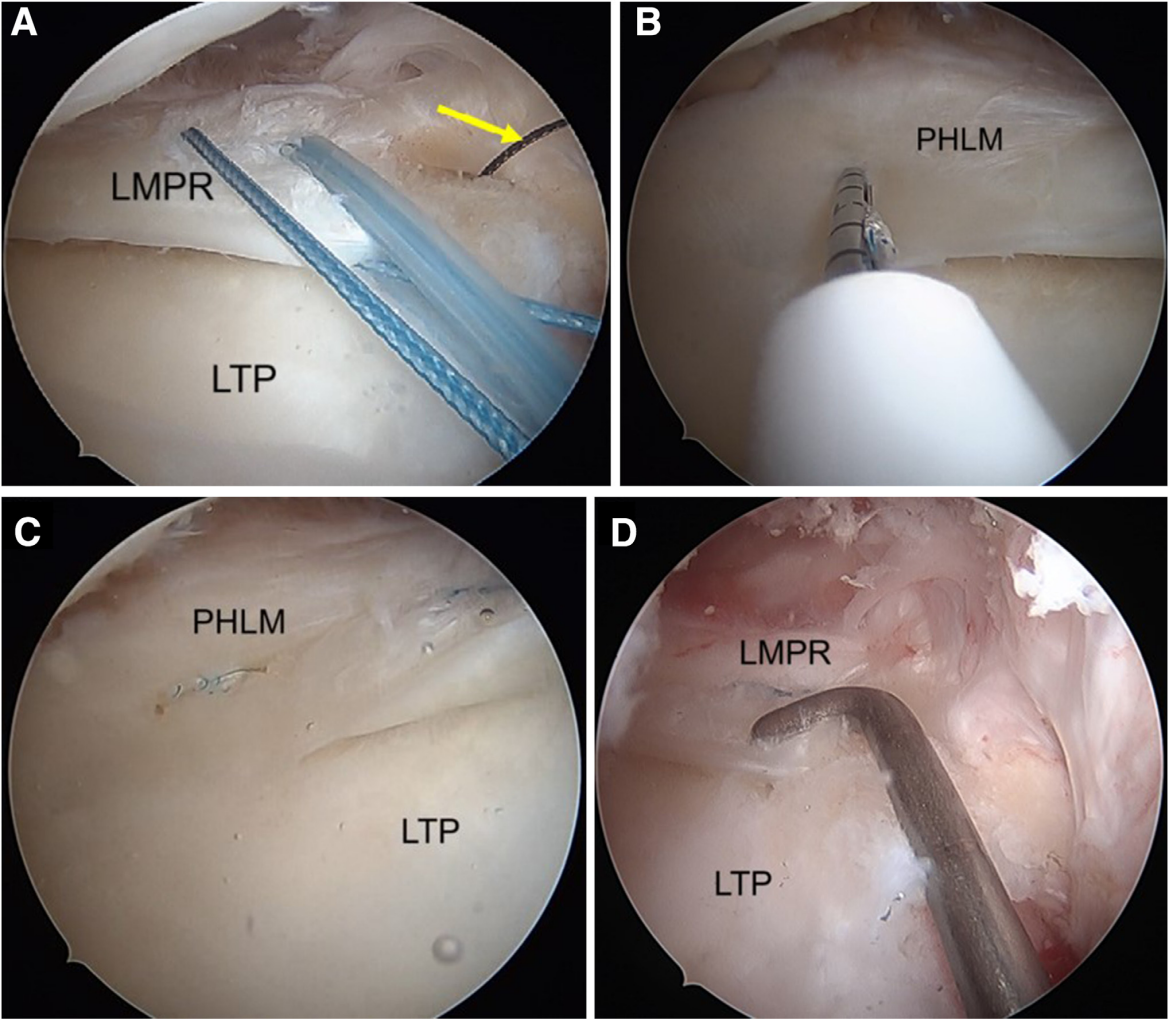
Arthroscopic pictures of right knee showing LMPR repair (patient in supine position, right limb in figure-of-four positions, view from anterolateral portal). (A) shows a shuttle suture (yellow arrow) through the tunnel of the lateral root. The tape and suture through the LMPR are pulled out through the tibial tunnel using a shuttle suture. (B) An all-inside meniscus repair device is used for placing additional security suture on the posterior horn of the lateral meniscus. (C) A horizontal suture configuration is used to secure the meniscus. (D) After fixation of the lateral meniscus root, the stability of the repair is checked using a probe. (LMPR, lateral meniscus posterior root; LTP, lateral tibial plateau; PHLM, posterior horn of lateral meniscus.)

The previous ACL tibial tunnel is first identified on the tibial externally and then the meniscal pull-out tunnel is planned medial to the ACL tunnel. A meniscal root guide is positioned at the native LMPR attachment (Fig 11C) at an angle of 50°, and a guidewire is inserted with its position confirmed arthroscopically (Fig 11B). This is followed by tunnel reaming using a 4.5-mm cannulated drill bit (Fig 11C). A shuttle relay wire is passed through the tunnel and is used to retrieve the tape and suture (Fig 12A). Tensioning the suture and tape reduces the lateral meniscus root into its tibial footprint.

A curved FAST-FIX 360 (Smith & Nephew) is used to repair the meniscocapsular separation of the posterior lateral meniscus (Fig 12B). This secures the posterior horn to the adjacent posterior capsule, which adds stability and reduces tension to the LMPR repair (Fig 12C). The tape and sutures are kept in this position and not secured till after the revision ACL tibia tunnel is reamed (Fig 12D).

### Revision ACLR and Lateral Extra-Articular Tenodesis (LET)

**Fig 13:**
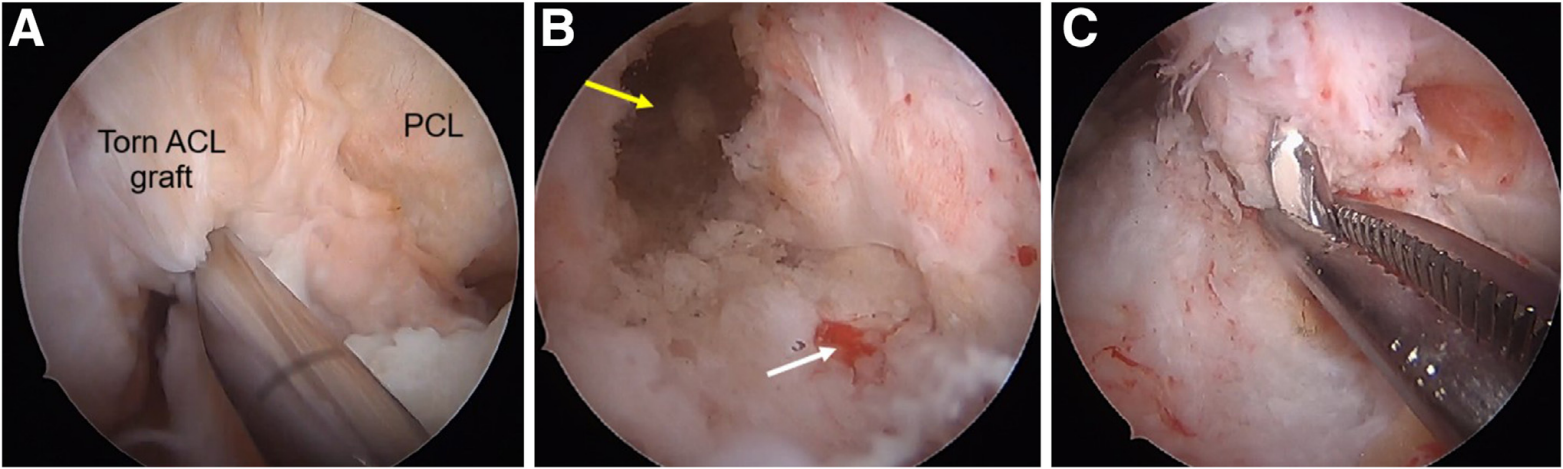
Arthroscopic pictures of right knee during the revision ACL reconstruction (patient in supine position, view from anterolateral portal). (A) The right knee notch view shows the torn ACL with remnant stump. (B) After preparation of the femoral tunnel (yellow arrow). Tibia previous tunnel can also be seen (white arrow). (C) Passage of the guidewire through the previous tibial tunnel. (ACL, anterior cruciate ligament; PCL, posterior cruciate ligament.)

**Fig 14:**
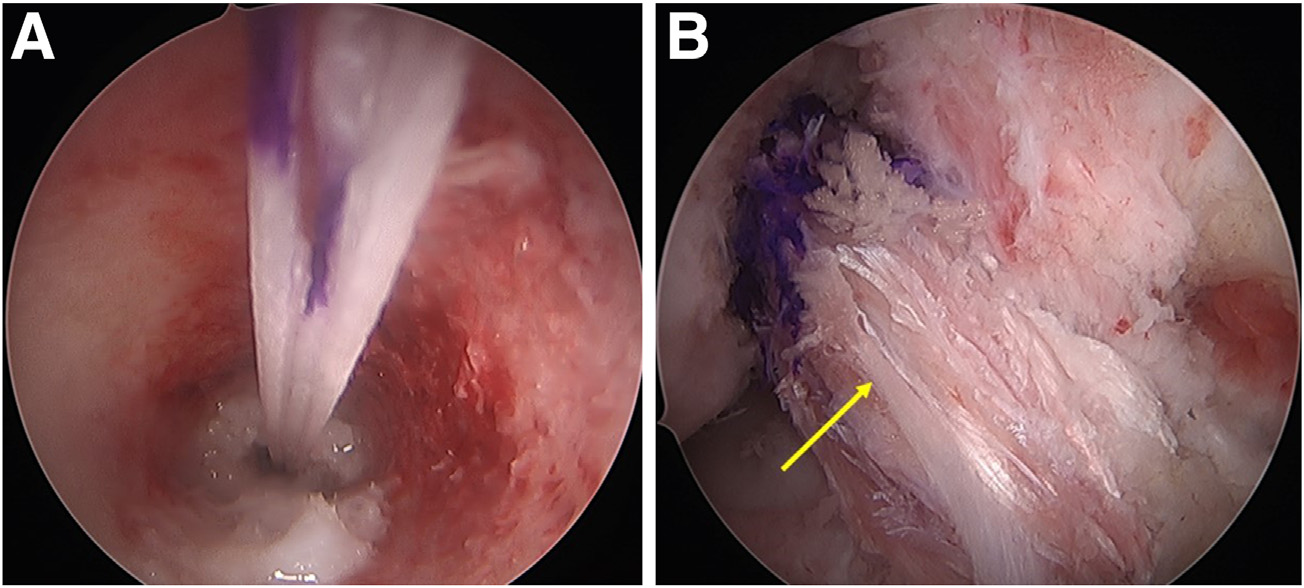
Arthroscopic pictures of right knee showing revision ACLR (patient in supine position). (A) The femoral tunnel after flipping the endo-button, viewed from the anteromedial portal. (B) The newly reconstructed anterior cruciate ligament (yellow arrow). (ACLR, anterior cruciate ligament reconstruction.)

The quadriceps autograft revision ACLR is performed using the previous tibial and femoral tunnels. In this patient, the previous tunnels were used, as they were deemed to be in an acceptable position (Fig 13 A-C). The root sutures are inspected during drilling of the tibia tunnels. After fixation of the revision ACL quadriceps graft (Fig 14 A and B), the lateral meniscus root tape and suture are fixed using a knotless anchor footprint (Smith & Nephew) with the knee in 30° of knee flexion.

**Fig 15:**
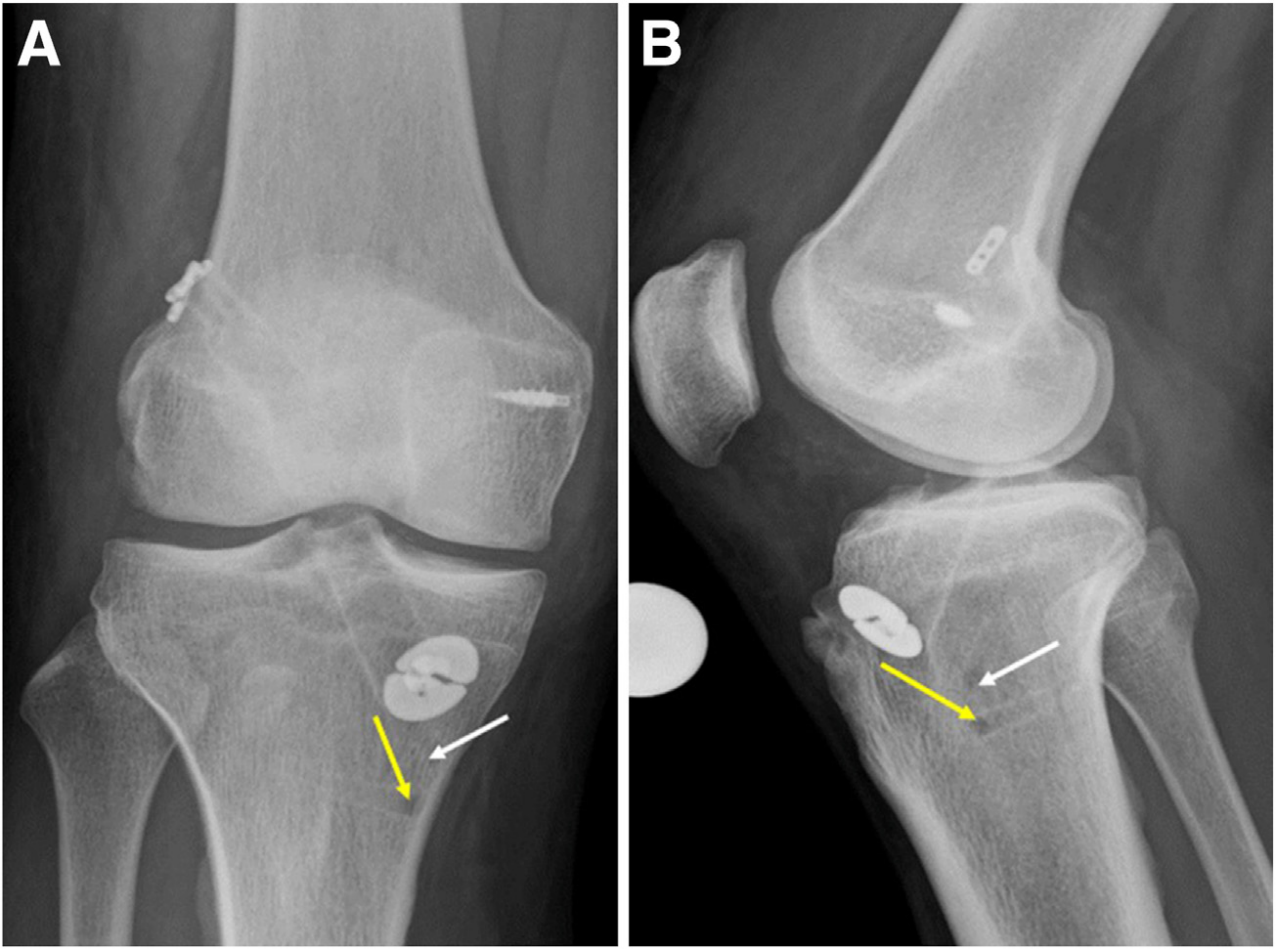
Postoperative radiographs of right knee of patient with new terrible triad with revision ACL reconstruction. (A) Anteroposterior view. A metallic anchor was used for MCL repair during previous surgery. (B) Lateral view. The lateral meniscus root tunnel depicted by white arrow. The root sutures are secured with a suture anchor distally (yellow arrow). The reconstructed ACL graft is fixed with a button. (ACL, anterior cruciate ligament; MCL, medial collateral ligament.)

We then proceed to perform the LET using the modified deep Lemaire technique. The postoperative radiograph shows the good position of the ACL and lateral meniscus root repair tunnels (Fig 15).

## Discussion

MMRLs and LMPRTs often are missed because of the low sensitivity of magnetic resonance imaging in detecting these lesions.^9^^,^^10^ There needs to be a high index of suspicion to the possibility of such lesions in patients who undergo revision ACL, especially those presenting with high-grade Lachman tests or explosive pivot shift tests. A thorough arthroscopic evaluation is essential to identify these meniscus injuries.^6^ In patients with ACL graft failure, it is possible that the combined MMRL and LMPRT that were unaddressed in the initial surgery could have contributed to persistent knee instability after ACLR and subsequent ACL graft failure.

MMRL involves tears at the medial meniscus meniscocapsular or meniscotibial attachments. These are sometimes difficult to detect via standard anterior arthroscopic portals, without additional assistance.^11^ Two other arthroscopic approaches have been described to improve detecting MMRLs: viewing through the intercondylar notch and the posteromedial portal approach. Malatray et al.^12^ reported that posteromedial portal viewing was not significantly better than the intercondylar approach. In our case, pie-crusting the MCL improves MMRL visualization from the anterior portals. This is often combined with viewing the meniscocapsular junction through the intercondylar notch.

The repair techniques for MMRL include inside-out suture repair with posteromedial safety incisions,^10^ all-inside meniscus suture devices,^13^ retrograde suture-passing devices with all suture repair,^14^^,^^15^ and tibial suture anchors.^16^ The use of suture hook repair and all suture repair through single or dual posteromedial portals has been recommended for MMRL.^14^^,^^15^ Saint-Etienne et al.^17^ compared all-inside meniscus repair from the anterior approach versus suture hook repair with posteromedial portals. They reported that the rates of subsequent surgery were the same for both techniques.

In our technique, we use all-inside medial meniscus ramp repair from anterior portals. It is important to be aware of the distances that the all-inside device is used to pierce the posterior capsule to avoid iatrogenic injury to the posterior knee vascular structures.^18^ This anterior approach of visualization and repair reduces surgical time and procedure complexity when multiple surgical procedures are required as compared with repair from posteromedial portals. This is important in complex cases in which 3 other surgical procedures have to be performed, namely LMPRT repair, revision ACLR, and LET.

The techniques described for LMPRT repair include side-to-side repair, suture anchor repair, and transtibial tunnel root repair. Transtibial meniscus root repair is the most common technique. It has been reported to achieve improved knee function, pain relief, and reduced meniscal extrusion.^19^

The transtibial tunnel technique can be done either by creating a single or double tunnel. Single-tunnel repairs are technically easier, especially when multiple tibial tunnels with ACLR are needed.^20^ This is even more pertinent in revision ACLR cases in which the tunnels can be enlarged and tibia bone stock can be limited. Therefore, it is critical to have careful planning to avoid tunnel convergence between the revision ACL tunnels and LMPR tibial tunnels.

A cadaver study by Campbell et al.^21^ showed LMPR tunnels drilled medially were closer to the ACL tunnel than those drilled laterally, favoring lateral tunnel placement to avoid tunnel collision with the ACL tibia tunnel. Chernchujit and Prasetia^22^ recommended placing tunnels from the anterolateral tibial side for both medial and lateral meniscus root tunnels when combined with ACLR.^22^ Shetty et al.^23^ advised arranging tibial tunnels from medial to lateral (medial meniscus root, lateral meniscus root, and ACL tibial tunnel), with all exits on the medial tibial surface.

**Fig 16:**
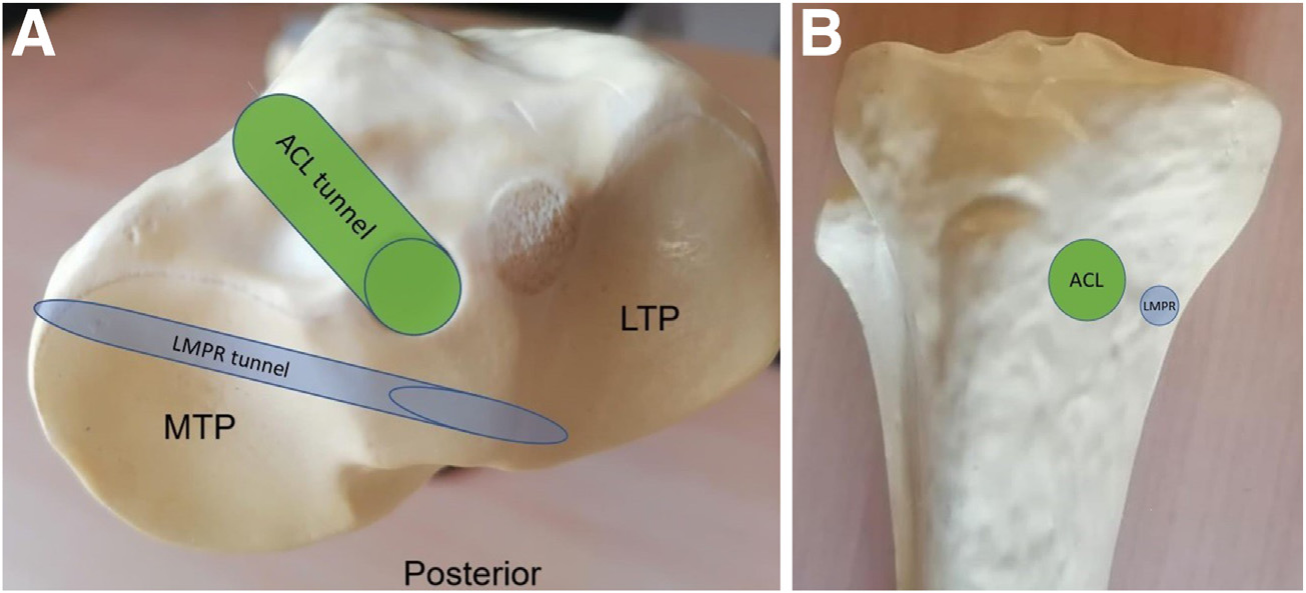
(A-B) A schematic illustration of right knee showing the arrangement of the lateral meniscus root repair tunnel and ACL tibial tunnel. Note adequate bony bridge between the tunnels. (ACL, anterior cruciate ligament; LMPR, lateral meniscus posterior root; LTP, lateral tibial plateau; MTP, medial tibial plateau.)

In our technique, the pull-out tunnel for LMPR is positioned medial and inferior to the revision ACL tunnel as described by Shetty et al.,^23^ thus maintaining the 2 tunnels in 2 different planes and avoiding tunnel convergence. Meniscus sutures are passed through the LMPR tunnel before the ACL tunnel is drilled, and no tunnel convergence is seen in our technique. As shown in the diagram, the more medial trajectory of the LMPR tunnel can avoid the ACL tunnel (Fig 16).

We supplement transtibial repairs with all-inside repairs to the posterior lateral capsule, these capsule repairs reinforce the LMPRT repair and offload the transtibial root repair site, which we believe reduces retears at the root junction and enhances outcomes.^24^ The tips and tricks of our technique as well as the advantages and disadvantages of our approach to manage MMRL and LMPRT are discussed in Table 1 and Table 2, respectively.

**Table 1:** Tips for Medial Meniscus Ramp Repair and Lateral Meniscus Posterior Root Repair

Medial Meniscus Ramp Repair	Lateral Meniscus Posterior Root Repair
Perform pie-crusting of MCL	A Figure-of-four position to open up the lateral compartment to minimize injury to the lateral compartment cartilage
Combine anterior arthroscopy with viewing from the intercondylar notch	Pass tape and sutures into good meniscus tissue near the meniscus capsule junction lateral meniscus
Achieve a balanced repair with sutures on the upper surface and lower surface of the medial meniscus	LMPR tibia tunnel is positioned medial to the ACL tunnel

ACL, anterior cruciate ligament; MCL, medial collateral ligament; LMPR, lateral meniscus posterior root.

**Table 2:** Advantages and Disadvantages of Single-Stage Revision ACLR With Associated MMRR and LMPRT repair

Procedure Steps	Advantages	Disadvantages	Risks
Single-staged procedure	Reduced patient costs, reduced stiffness, and early recovery	Longer operating time, need experienced surgeon to perform	Blood loss, prolonged torniquet time
Quadriceps autograft	High tensile strength, quicker harvest, easier fixation without risk of tunnel coalescence	Additional anterior skin incision, can delay postoperative recovery	Graft amputation
All-inside MMRR	Reduced surgical time and complexity	Adequate exposure by pie-crusting is crucial	Posterior neurovascular injury, MFC cartilage damage in case of poorly done MCL release
Single-tunnel transtibial for LMPRT	Technically less demanding, reduced surgery time, lower risk of tunnel collision	Smaller footprint area for root reinsertion compared with double tunnel technique	LFC cartilage damage while passing the instruments.
Onlay LET with anchor	Easier, faster, lower risk of tunnel coalescence	Placement of anchor in the presence of a femoral ACL tunnel that is in proximity leads to poor fixation stability	Anchor pull out due to poor bone strength.

LET, lateral extra-articular tenodesis; LMPRT, lateral meniscus posterior root tear; MFC, Medial femoral condyle; MMRL, medial meniscus ramp lesion; MMRR, medial meniscus ramp repair.

Saint-Etienne et al.^17^ showed that isolated ACLR without LET increased the risk of further surgery eightfold in the presence of meniscal ramp or root tears. The addition of the LET provides additional protection to the ACL graft. It also has been shown that revision ACLR with LET can improve rotational stability and reduce failure rates compared with revision ACLR alone.^25^ We add the LET for all revision ACLR patients for these reasons. We use quadriceps autograft for revision ACLR, and the evidence reports its results are comparable with bone patella bone tendon autograft.^26^

In summary, we outline a simple and systematic approach for managing combined MMRL and LMPRT during revision ACLR. With planning, surgeons can make use of this approach to streamline the practice, avoid intraoperative challenges, and improve patient outcomes with improved knee stability.

## Disclosures

All authors (S.S., S.A., Y.R.M., Y.H.D.L.) declare that they have no known competing financial interests or personal relationships that could have appeared to influence the work reported in this paper.

## Supplementary Data

Video 1 This surgical technique addresses concurrent ACL graft failure, MMRL, and LMPRT known as “the new terrible triad “ A high-grade Lachman or pivot shift test during clinical examination should prompt suspicion of these injuries. Preoperative imaging includes MRI to identify the lesions and computed tomography scan to assess previous ACL tunnel sizes. The procedure is done under general anesthesia with operative limb placed in hanging knee position. A near full-thickness quadriceps graft is harvested. A diagnostic arthroscopy is performed with the standard anterolateral and anteromedial portals. Upon identification of the MMRL, the medial meniscus ramp lesion is rasped and then repaired using an all-inside repair device. The first stitch is placed in the meniscus tissue from the superior surface of the meniscus and the second stitch is placed in the capsule, stabilizing meniscocapsular junction. This is repeated in inferior surface of the meniscus to repair the meniscotibial ligaments. The LMPRT is repaired with a single-tunnel pull-out technique. After preparing the lateral meniscus root footprint, a tape and a suture are placed in robust meniscal root tissue using an antegrade suture passage device. This tunnel is drilled medial to the previous ACL tunnel in the tibia to minimize tunnel collision. The tape and suture are passed into the lateral root tunnel. This meniscus root repair is supplemented by an all-inside meniscus repair stitch in the posterior horn. The previous femoral and tibial ACL tunnels are identified and prepared. The ACL graft is passed and secured with cortical fixation in both the femur and the tibia. The lateral root tape and sutures are secured on the tibia with a knotless anchor. LET is performed using a modified Lemaire technique. (ACL, anterior cruciate ligament; CT, computed tomography; LET, lateral extra-articular tenodesis; LMPR, lateral meniscus posterior root; LMPRT, lateral meniscus posterior root tear; LTP, lateral tibial plateau; MMRL, medial meniscus ramp lesion; MTP, medial tibial plateau.)
